# Impact of Selective Evidence Presentation on Judgments of Health Inequality Trends: An Experimental Study

**DOI:** 10.1371/journal.pone.0063362

**Published:** 2013-05-16

**Authors:** Sam Harper, Nicholas B. King, Meredith E. Young

**Affiliations:** 1 Department of Epidemiology, Biostatistics and Occupational Health, McGill University, Montreal, Quebec, Canada; 2 Department of Social Studies of Medicine, McGill University, Montreal, Quebec, Canada; 3 Biomedical Ethics Unit, McGill University, Montreal, Quebec, Canada; 4 Centre for Medical Education, McGill University, Montreal, Quebec, Canada; 5 Department of Medicine, McGill University, Montreal, Quebec, Canada; Universität Bochum, Germany

## Abstract

Reducing health inequalities is a key objective for many governments and public health organizations. Whether inequalities are measured on the absolute (difference) or relative (ratio) scale can have a significant impact on judgments about whether health inequalities are increasing or decreasing, but both of these measures are not often presented in empirical studies. In this study we investigated the impact of selective presentation of health inequality measures on judgments of health inequality trends among 40 university undergraduates. We randomized participants to see either a difference or ratio measure of health inequality alongside raw mortality rates in 5 different scenarios. At baseline there were no differences between treatment groups in assessments of inequality trends, but selective exposure to the same raw data augmented with ratio versus difference inequality graphs altered participants’ assessments of inequality change. When absolute inequality decreased and relative inequality increased, exposure to ratio measures increased the probability of concluding that inequality had increased from 32.5% to 70%, but exposure to difference measures did not (35% vs. 25%). Selective exposure to ratio versus difference inequality graphs thus increased the difference between groups in concluding that inequality had increased from 2.5% (95% CI −9.5% to 14.5%) to 45% (95% CI 29.4 to 60.6). A similar pattern was evident for other scenarios where absolute and relative inequality trends gave conflicting results. In cases where measures of absolute and relative inequality both increased or both decreased, we did not find any evidence that assignment to ratio vs. difference graphs had an impact on assessments of inequality change. Selective reporting of measures of health inequality has the potential to create biased judgments of progress in ameliorating health inequalities.

## Introduction

Reducing health inequalities is an important public health policy goal in many wealthy countries [1], [2]. However, measuring progress towards reducing health inequalities is rarely straightforward and involves a number of methodological considerations, including choice of reference points for measuring departures from equality, considerations of the size of social groups, and whether inequalities are expressed on the absolute or relative scale [3]–[5].

Whether inequalities are measured on the absolute (difference) or relative (ratio) scale can have a significant impact on judgments about whether health inequalities are increasing or decreasing [6]–[8]. In cases where rates of disease are declining for all groups, absolute and relative measures of inequality will diverge if the relative but not the absolute rate of decline is greater among the better off (i.e., healthier) group [6]. This suggests that empirical studies of health inequality trends that present only absolute or relative measures of inequality could bias judgments regarding progress in reducing inequalities. While reviews of measuring health inequalities [3], [9] typically recommend presenting both absolute and relative measures of inequality (consistent with the STrengthening the Reporting of OBservational Epidemiology (STROBE) guidelines [10]), many studies still rely exclusively on relative inequality measures [11]–[16].

Concerns about the scale of measures of health inequalities parallel those raised in the context of absolute and relative risks in clinical epidemiology. Previous research demonstrates that absolute risks are often not available in the abstracts or full text of reports of many clinical trials [17], and that health professionals’ clinical decisions are influenced by the presentation of risks in absolute or relative terms [18]. However, to date there is little evidence on whether the selective presentation of absolute or relative health inequality measures influences judgments about the magnitude of health inequalities, or willingness to support health interventions. We used an experimental design to test whether judgments of the impacts of interventions on health inequalities may be influenced by the selective presentation of absolute or relative health inequality measures.

## Methods

### Participants

We recruited 40 students enrolled at McGill University (30 female, age range 17–37) to participate in this study in exchange for a $15 honorarium. Participants were recruited from on-line classified postings, were not restricted by course of study, and the only inclusion criterion was an ability to speak English with near-native fluency. Participants were orally briefed regarding the procedures of the experiment, and written consent was obtained from all participants. As this study involved minimal risk, per the Canadian *Tri-Council Policy Statement: Ethical Conduct for Research Involving Humans* [19], we did not obtain additional consent from a caretaker, guardian, or next of kin on behalf of the 17-year old university student who participated in this study. The McGill University Faculty of Medicine Institutional Review Board approved this study, including the consent procedure, for university students of all ages.

### Study Design

Upon arrival, participants were orally briefed regarding the procedures of the experiment, and written consent was obtained. Participants were asked to complete a custom computer-based task programmed using LiveCode (2009), in which they were asked to evaluate the impact of an intervention on the health of two populations. We used generic populations (“population A” and “population B”) and fictional diseases [20] in order to limit the influence of participants’ previous experience or information.

**Figure 1:**
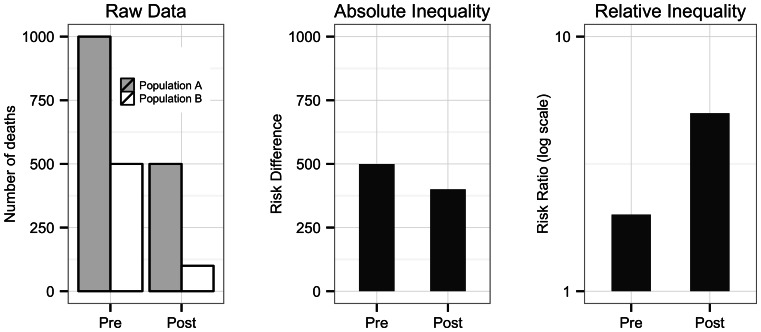
Example of an inconsistent scenario with decreasing absolute and increasing relative inequalities.

The present study was part of larger study of cognitive bias in the evaluation of health inequalities. In this study we presented individuals with graphical data on health inequalities in mortality (i.e. how many individuals to a maximum of 1,000 died) before and after a health intervention. For a given health intervention scenario, each of the 40 participants were randomized into one of two treatment arms, “difference” (n = 20) or “ratio” (n = 20). At Time 1 (pre-treatment) all participants were presented with “Raw Data”: a single graph that displayed mortality rates for two populations (*A* and *B*) before and after an unspecified health intervention. At Time 2 (post-treatment) the “difference” group was shown the same “Raw Data” graph plus a graph that displayed a measure of absolute inequality (rate difference, Population *A* − *B*). Similarly, the “ratio” group was shown the “Raw Data” plus a graph that displayed a measure of relative inequality (rate ratio, Population *A* ÷ Population *B*). Figure 1 shows an example of the graphs used for a scenario where absolute inequality decreases but relative inequality increases. The order of the scenarios was randomized across participants, as were the fictional diseases. We hypothesized that the inclusion of either a difference or a ratio measure of inequality would affect respondents’ judgments about whether inequality between the two groups increased or decreased.

We created 10 scenarios: 5 sets of 2 scenarios each. In 3 sets of scenarios absolute and relative measures were “inconsistent” – i.e., they disagreed with respect to whether inequality was increasing, decreasing, or staying constant (decreasing absolute inequality and increasing relative inequality; constant absolute inequality and increasing relative inequality; decreasing absolute inequality and constant relative inequality). In the other 2 sets of scenarios, absolute and relative measures were “consistent” – i.e., they agreed with respect to whether inequality was increasing, decreasing, or staying constant (both increasing; both decreasing). Within each scenario set, we varied the magnitude of the effect of the intervention to assess whether participant judgments were sensitive to smaller vs. larger changes in inequality. Table S1 lists the hypothetical mortality values and measures of relative and absolute inequality for all 10 scenarios.

For each scenario, at both Time 1 and Time 2 participants were asked to indicate their judgment regarding the following statement: “The inequality between population A and population B has: (a) decreased, (b) increased, (c) stayed the same, (d) don’t know.” Participants were also asked: 2) “How successful was the program in reducing inequality between population A and population B?” (7-point scale where 1 = not at all successful, and 7 = very successful); 3) “A $100 tax increase has been approved to fund various health initiatives, however how that increase will be used is still under debate. Remember that if you choose to dedicate some (or all) of that $100 to this health intervention, it will not be available for other interventions. So, of that $100 increase, how much would you dedicate to support the continuation of this health intervention?” (100 point scale from $0–100); and 4) “Should this intervention continue?” (7-point scale where 1 = Should definitely not continue, and 7 = Very definitely should continue).

### Statistical Analysis

We used chi-square tests and regression to analyze the impact of selective exposure to graphs containing measures of absolute vs. relative inequality. For chi-square tests where expected cell sizes were less than 5 we used Fisher’s exact significance test. For the main question of how inequality changed after the intervention we used logistic regression and defined the outcome as the response that was consistent with the measure of relative inequality. That is, for the scenarios where the ratio increased we estimated the likelihood of concluding that inequality had increased vs. any other response; for scenarios where the ratio decreased we estimated the likelihood of concluding that inequality had decreased vs. any other response; and similarly for scenarios where the ratio was constant. For respondent assessments of program success, willingness to donate money, and whether the intervention should continue we used linear regression. Because for each the 5 scenarios we also varied the magnitude of the intervention effect, in all the regression analyses we tested whether the treatment effect differed by the magnitude of the change in inequality (larger vs. smaller) by including a product term between treatment and the scenario variation in magnitude. For the logistic regression analysis we tested for heterogeneity on the absolute probability scale [21]. If there was evidence of a differential treatment effect by scenario magnitude we estimated separate treatment effects for larger vs. smaller inequality changes; if not, we pooled the larger and smaller scenarios and adjusted for magnitude using an indicator variable. Finally, using regression models adjusted for scenario magnitude we estimated marginal effects of the treatment on the probability of agreement with the relative inequality measure, holding constant scenario magnitude. All analyses were conducted using Stata 12 software (StataCorp, College Station, TX).

## Results

Table 1 shows the proportion of individual assessments of the post-intervention change in inequality for both treatment arms at Time 1 (raw data graph only), and at Time 2 (raw data graph plus a graph with an inequality measure). Chi-square tests for equality of proportions are also shown for differences between treatment groups. At Time 1 (raw data only) there were no differences across treatment groups in the proportion concluding that inequality had decreased, increased, or stayed the same. At Time 2 (raw data plus inequality graphs) assignment to seeing either difference or ratio measures of inequality alongside the raw data affected individuals’ judgments of the direction of change in health inequality for the 3 inconsistent scenarios, but there was little variation by treatment arm for the 2 consistent scenarios. Table 1 also shows some evidence that in cases of inconsistent relative and absolute inequality trends individual assessments (regardless of treatment) tend to reflect absolute inequality trends. For example, with decreasing absolute and increasing relative inequality, roughly 60% of both treatment groups concluded that inequality had decreased when viewing only the raw data.

**Table 1:** Impact of presenting absolute vs. relative inequality graphs in addition to baseline rates on judgments of the impact of a hypothetical intervention on inequality trends.

			Raw data only at Time 1				Inequality graph at Time 2			
			Treatment Group				Treatment Group			
Inequality Scenario			Difference		Ratio		Difference		Ratio	
Difference	Ratio	Respondent Assessment	No.	%	No.	%	No.	%	No.	%
Decrease	Increase	Decreased	24	60.0	23	57.5	27	67.5	9	22.5
		Increased	13	32.5	14	35.0	10	25.0	28	70.0
		Same	3	7.5	2	5.0	1	2.5	2	5.0
		Don’t know	0	0.0	1	2.5	2	5.0	1	2.5
		Total	40	100.0	40	100.0	40	100.0	40	100.0
			?^2^ = 1.26, *p = *1.0				?^2^ = 18.19, *p*<0.001			
Constant	Increase	Decreased	6	15.0	1	2.5	2	5.0	0	0.0
		Increased	14	35.0	12	30.0	14	35.0	26	65.0
		Same	19	47.5	27	67.5	23	57.5	12	30.0
		Don’t know	1	2.5	0	0.0	1	2.5	2	5.0
		Total	40	100.0	40	100.0	40	100.0	40	100.0
			?^2^ = 6.12, *p* = 0.070				?^2^ = 9.39, *p* = 0.011			
Decrease	Constant	Decreased	21	52.5	19	47.5	25	62.5	7	17.5
		Increased	2	5.0	3	7.5	1	2.5	0	0
		Same	17	42.5	17	42.5	13	32.5	33	82.5
		Don’t know	0	0.0	1	2.5	1	2.5	0	0
		Total	40	100.0	40	100.0	40	100.0	40	100.0
			?^2^ = 1.30, *p* = 0.940				?^2^ = 20.82, *p*<0.001			
Decrease	Decrease	Decreased	32	80.0	36	90.0	38	95.0	38	95.0
		Increased	3	7.5	4	10.0	1	2.5	2	5.0
		Same	3	7.5	0	0.0	1	2.5	0	0
		Don’t know	2	5.0	0	0.0	0	0.0	0	0
		Total	40	100.0	40	100.0	40	100.0	40	100.0
			?^2^ = 5.38, p = 0.141				?^2^ = 1.33, p = 1.0			
Increase	Increase	Decreased	4	10.0	3	7.5	3	7.5	3	7.5
		Increased	36	90.0	36	90.0	37	92.5	37	92.5
		Same	0	0.0	1	2.5	0	0.0	0	0.0
		Don’t know	0	0.0	0	0.0	0	0.0	0	0.0
		Total	40	100.0	40	100.0	40	100.0	40	100.0
			?^2^ = 1.14, p = 1.0				?^2^ = 0.00, p = 1.0			

Note: Chi-square test is for difference of proportions across treatment groups. Fisher’s exact p-value. N = 20 for each treatment group, but each panel shows the total sample pooled across the magnitude of change (large vs. small change in inequality) for each Inequality Scenario.

For judgments of inequality change we found no evidence of treatment heterogeneity by scenario magnitude, with *p*-values of 0.236, 0.152, and 0.400 for the 3 inconsistent inequality scenarios, and 0.573 and 0.471 for the 2 consistent inequality scenarios. Thus we pooled across larger and smaller magnitudes of inequality changes, and adjusted for magnitude using regression.

**Figure 2:**
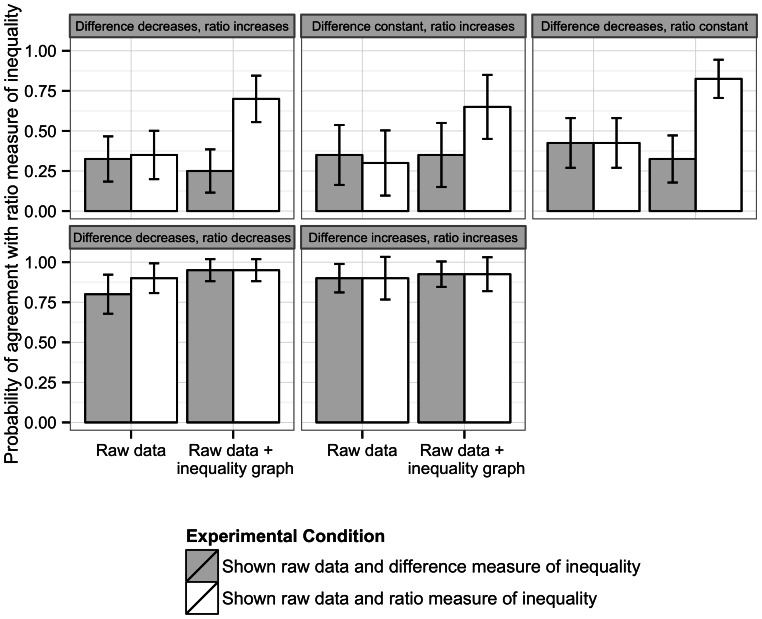
Impact of including a difference or ratio measure of inequality alongside raw data on the judgment of inequality trends after a hypothetical intervention.

Figure 2 shows, for each set of scenarios, marginal estimates from logistic regression models of the probability of the respondent agreeing with the ratio measure of inequality, holding constant the magnitude of the inequality change. For the first set of inconsistent scenarios, in which absolute inequalities declined but relative inequalities increased, individuals randomized to be shown graphs including ratio or difference measures of inequality did not differ in their assessments of whether inequalities had increased when shown only the raw data (32.5% vs. 35.0% at pre-treatment). After exposure to scenarios that included both the raw data and graphs displaying a ratio measure of inequality, the proportion of individuals concluding that inequality had increased rose to 70% (95% confidence interval (CI): 55.5 to 84.5). In contrast, among those exposed to the same raw data and a difference measure of inequality, the proportion concluding inequality had increased fell to 25% (95% CI 11.5 to 38.5), which was not statistically distinguishable from their pre-treatment assessment. Selective exposure to ratio versus difference inequality graphs thus increased the difference between groups in concluding that inequality had increased from 2.5% (95% CI −9.5% to 14.5%) to 45% (95% CI 29.4 to 60.6).

For scenarios in which absolute inequality remained constant, while relative inequality increased, we found a similar differential effect between presenting difference versus ratio measures of inequality (Figure 2). Exposure to difference measures had little impact on participants’ estimations of whether inequality increased, but exposure to ratio inequality measures increased the proportion concluding that inequalities had increased from 30% to 65%, and increased the difference between treatment groups in concluding inequality had increased from −5% to 30% (95% CI 1.8% to 22.6%).

We found a similar pattern for scenarios in which absolute inequality decreased and relative inequality remained constant (Figure 2). When exposed to the raw data only (pre-treatment), 42.5% of both treatment groups concluded that inequalities had stayed the same. However, exposure to both raw data and a ratio measure of inequality (post-treatment) increased this percentage to 82.5% (95% CI 70.6 to 94.4), whereas exposure to raw data and a measure of absolute inequality had little impact (32.5%, 95% CI 17.8 to 47.2).

In contrast to the above patterns for scenarios with conflicting absolute and relative inequalities, scenarios in which both relative and absolute inequalities changed in a consistent direction (both increasing or both decreasing) produced no evidence of differences between treatment groups.

In supplementary analyses of the other three outcomes we also found some evidence that, for scenarios where absolute and relative inequality trends were inconsistent, selective presentation of ratio vs. difference graphs altered individuals’ assessments of the impact of the hypothetical intervention (Figures S1, S2, S3). For example, for scenarios where absolute inequality decreased but relative inequality increased selective exposure to ratio measures of inequality decreased individuals’ assessments of program success by 0.88 points (7 point scale, 95% CI 0.03 to 1.72, Figure S1). A similar decrease in assessments of program success was seen for scenarios where absolute inequality decreased but relative inequality was constant (1.53 points, 95% CI 0.67 to 2.39). Exposure to ratio measures of inequality also suggested some impact on decreasing the amount of money respondents would pay to continue the intervention (Figure S2) and decreasing their likelihood of concluding that the intervention should continue (Figure S3). Regression estimates for all four outcomes are provided in Tables S2, S3, S4, S5.

## Discussion

In this study we experimentally manipulated the presentation of evidence on health inequalities in order to determine whether selective presentation of the same underlying data in exclusively absolute or relative terms might influence individual assessments of inequality trends. When presented with only raw data on the mortality rates of two populations before and after a hypothetical health intervention, individuals’ estimations tended to be more consistent with the absolute measure of inequality. In cases where the absolute and relative measures changed in a consistent direction (either both increasing or both decreasing), showing them an absolute or relative inequality measure alongside the raw data had no differential impact on their assessments. However, in cases where the inequality measures were inconsistent individuals shown a relative inequality measure were more likely to alter their estimation of the inequality trend than those shown an absolute inequality measure.

To our knowledge this is the first study to experimentally demonstrate the impact of presenting selective measures of health inequality trends on judgments about the direction of change in health inequalities. However, a number of studies in other domains have suggested that selective presentation of evidence may have important consequences on the interpretation of results. In clinical epidemiology, for example, several studies have shown that presentation of relative risk reductions in the absence of data on underlying absolute probabilities leads to systematic overestimation of risks and/or benefits [18], [22]. In one study, 57% of patients opted for a medication when its benefits were presented in relative terms, versus 15% when presented in absolute terms [23]. Clinicians were also more likely to report that they would be inclined to treat patients, and to rate an intervention effective, when presented with information in relative rather than in absolute terms [24], [25].

Our results have implications for both producers and consumers of evidence on the magnitude of health inequalities. Selective use of exclusively absolute or relative measures, particularly in cases where these measures conflict, may lead to biased assessments of whether inequalities are increasing or decreasing, or of which social groups or health outcomes may demonstrate the largest health inequalities [6], [15]. Prior work has shown that substantive judgments about health inequalities may be dramatically different depending on the choice of inequality measure. For example, Keppel ranked nearly 500 diseases in the United States according to the level of inequality across race-ethnic groups [15]. For conditions measured on different scales relative measures may be the only choice, but across outcomes on similar scales such as mortality rates, outcomes with relatively small absolute risks (e.g., drug-induced deaths) ranked highly, whereas those with greater absolute risks (e.g., heart disease) failed to make the top 10.

Selective presentation of evidence may also have an impact on measuring changes in health inequalities, either for general monitoring over time or when evaluating the effects of interventions on health inequalities. In these cases, there are many examples where absolute and relative inequalities disagree. This has been demonstrated in the context of secular trends in social class gradients in mortality [26], [27], measuring progress toward the Millenium Development Goals [28], racial [11], [29] and socioeconomic inequalities [14], [29]–[31] in the impact of highly active antiretroviral therapy on HIV mortality, and the impact of traditional risk factors on socioeconomic and gender differences in cardiovascular disease [32], [33], among others.

Our results reinforce existing guidelines recommending that researchers studying health inequalities present, when feasible, measures of inequality on both the absolute and relative scale [3], [9], [34]. This is in keeping with general guidelines for reporting the results of clinical trials and observational studies in epidemiology. In the same way that presentation of absolute risks alongside relative benefits may help patients and clinicians better decide among alternative treatments, presentation of absolute levels of disease and measures of absolute inequality may help put relative inequalities in context for policymakers and the public. Additionally, presentation of both absolute and relative inequalities, or at the very least explicit discussion of the issue of scale, may help researchers avoid accusations of bias or presentation of misleading evidence. For example, in commenting on the large number of policy documents on health inequalities published in the UK during the 1990s, Oliver, Healey, and Le Grand expressed a particular concern that the evidence on health inequalities was being “selectively reported so as to have a greater effect,” largely through the exclusive presentation of relative effects [35].

Finally, there is evidence that medical and public health literature exhibits a bias towards presenting empirical evidence of social inequalities in health in exclusively relative terms [16]. Given our findings, it is possible that consumers of medical and public health literature may be making systematically biased assessments of the magnitude, direction, and significance of social inequalities in health. This may, in turn, impact support for health interventions and public health policies.

Our study has limitations, the most notable being the restriction of our subjects to university undergraduates. Our study population was diverse in terms of courses of study, and predominantly composed of women. Having achieved admission to university, our population may be somewhat more numerate than the general population. Given the experimental design, characteristics of the study population are unlikely to create bias in our treatment effects, but it may limit the ability to generalize the results more widely. Our study also focused exclusively on scenarios for which all social groups experience improvements in mortality after our hypothetical intervention. Hypothetical interventions that may adversely affect health could produce different findings.

In conclusion, we found evidence that the selective presentation of a single measure of either absolute or relative inequality may substantially alter judgments about whether interventions reduce or exacerbate health inequalities. Researchers should follow existing recommendations to report both absolute and relative inequality measures whenever possible. Consumers of health inequalities research should be aware of the potential impact of selective reporting of health inequalities measures in absolute or relative terms.

## Supporting Information

Figure S1 Impact of presenting a difference or ratio measure of inequality alongside raw data on respondent’s judgment of success of a hypothetical intervention.
(TIFF)

Figure S2 Impact of presenting a difference or ratio measure of inequality alongside raw data on amount of respondent’s money for continuation of a hypothetical intervention.
(TIFF)

Figure S3 Impact of presenting a difference or ratio measure of inequality alongside raw data on whether a hypothetical intervention should continue.
(TIFF)

Table S1 Scenarios depicting inconsistent and consistent changes in absolute and relative mortality inequalities after a hypothetical intervention.
(PDF)

Table S2 Logistic regression estimates for judgment of whether inequality increased or decreased.
(PDF)

Table S3 Linear regression estimates for respondent’s judgment of program success.
(PDF)

Table S4 Regression estimates for amount of money respondent was willing to give to support continuation of the hypothetical program.
(PDF)

Table S5 Regression estimates for respondent’s assessment of whether the hypothetical program should continue.
(PDF)
