# Supplementary figures and images for: Impact of Selective Evidence Presentation on Judgments of Health Inequality Trends: An Experimental Study
Source: PLoS One. 2013 May 16;8(5):e63362. doi: 10.1371/journal.pone.0063362 (PMC3656043; doi:10.1371/journal.pone.0063362)

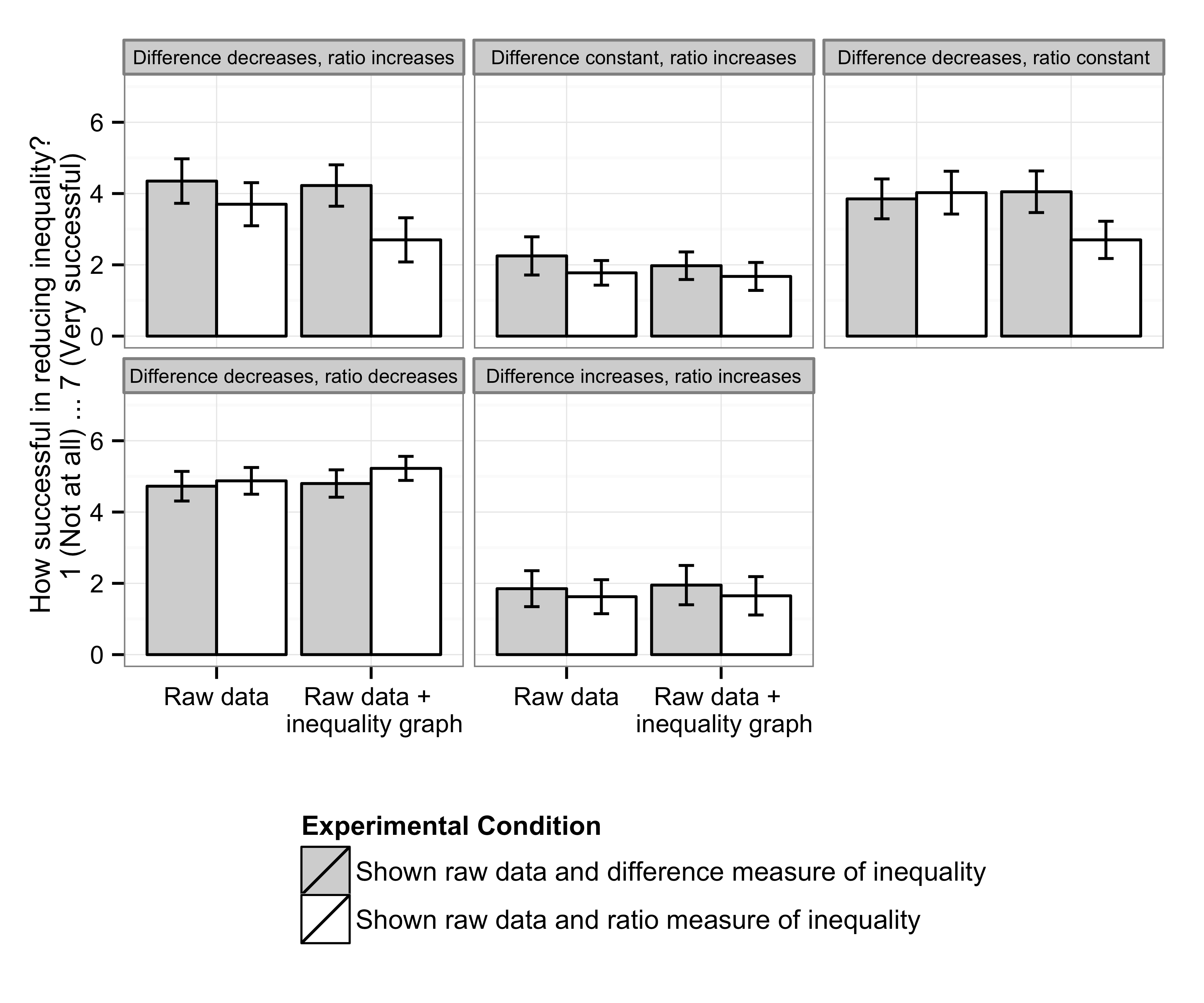

Supplement: Figure S1 — Impact of presenting a difference or ratio measure of inequality alongside raw data on respondent’s judgment of success of a hypothetical intervention. (TIFF) [file pone.0063362.s001.tiff]

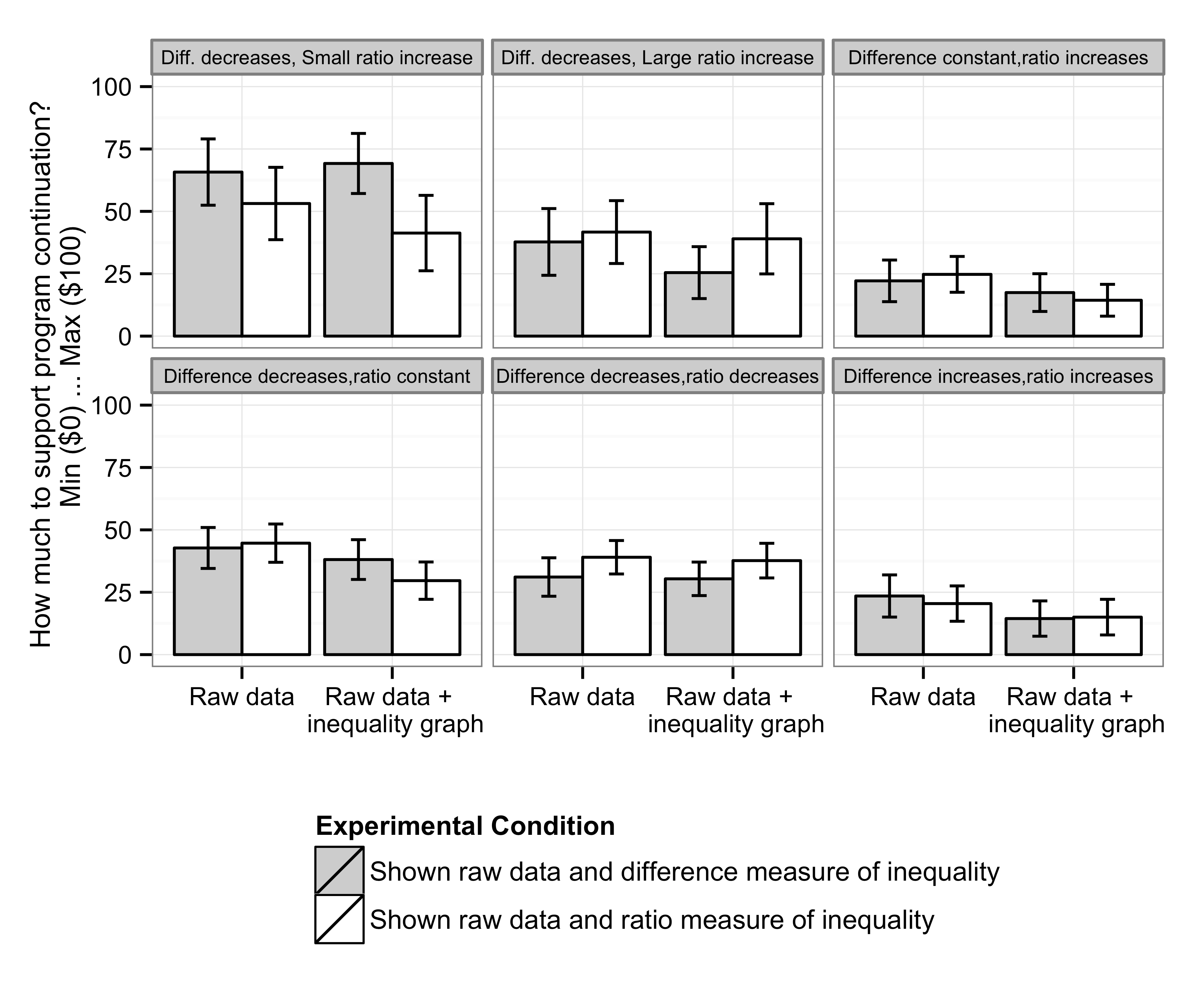

Supplement: Figure S2 — Impact of presenting a difference or ratio measure of inequality alongside raw data on amount of respondent’s money for continuation of a hypothetical intervention. (TIFF) [file pone.0063362.s002.tiff]

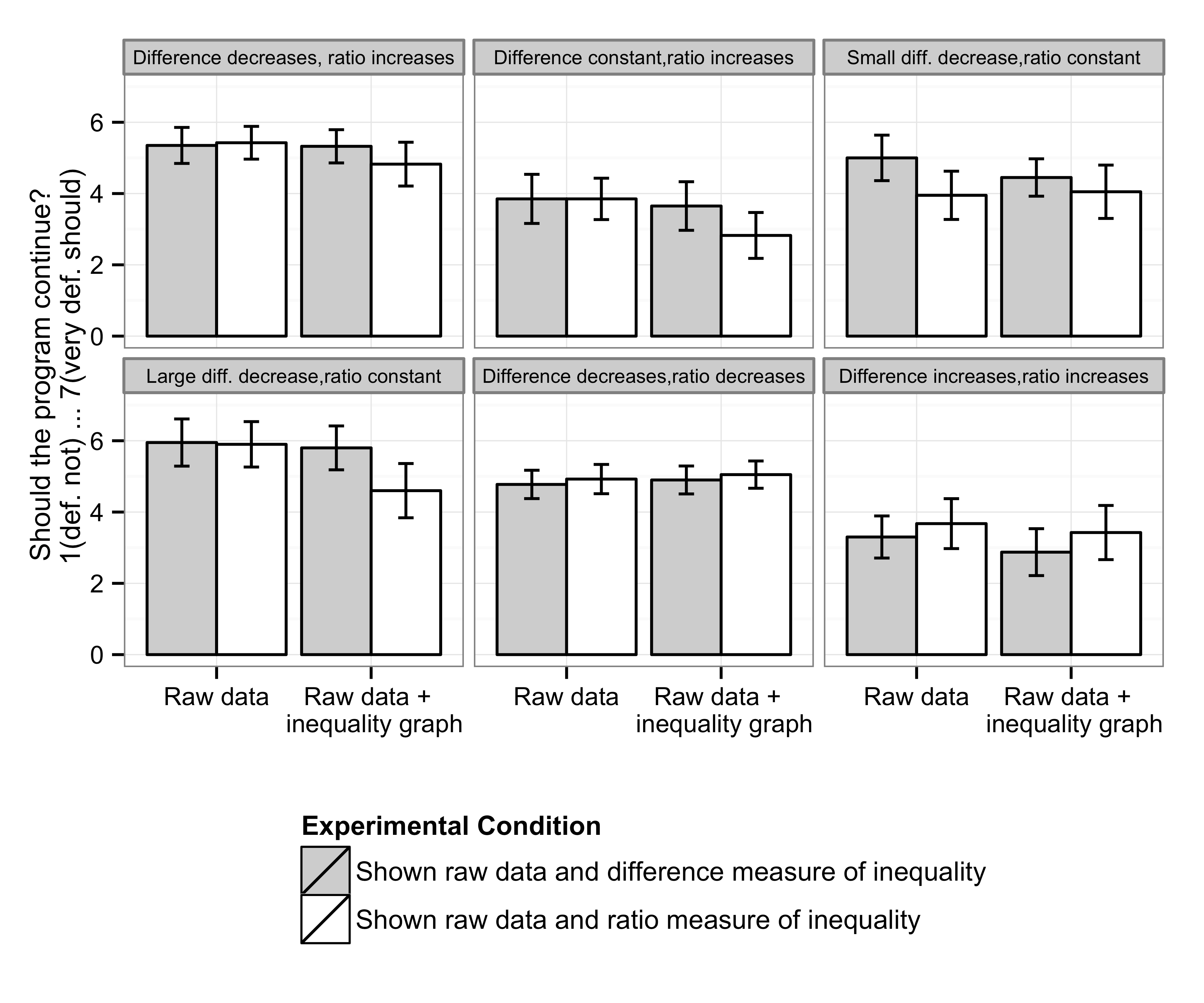

Supplement: Figure S3 — Impact of presenting a difference or ratio measure of inequality alongside raw data on whether a hypothetical intervention should continue. (TIFF) [file pone.0063362.s003.tiff]
