# Supplementary material for: Impact of Selective Evidence Presentation on Judgments of Health Inequality Trends: An Experimental Study
Source: PLoS One. 2013 May 16;8(5):e63362. doi: 10.1371/journal.pone.0063362 (PMC3656043; doi:10.1371/journal.pone.0063362)
Supplement: Table S1 — Scenarios depicting inconsistent and consistent changes in absolute and relative mortality inequalities after a hypothetical intervention. (PDF) [file pone.0063362.s004.pdf]

Table S1: Scenarios depicting inconsistent and consistent changes in absolute and relative mortality inequalities after a hypothetical intervention.

	Mortality Before Intervention		Mortality After Intervention		Inequality Before Intervention		Inequality After Intervention	
Scenario and magnitude of inequality change	Pop A	Pop B	Pop A	Pop B	Risk Diff.	Risk Ratio	Risk Diff.	Risk Ratio
Inconsistent: Decreasing absolute inequalities, increasing relative inequalities								
a) smaller change	1000	500	200	95	500	2.00	105	2.11
b) larger change	1000	500	500	100	500	2.00	400	5.00
Inconsistent: Constant absolute inequalities, increasing relative inequalities								
a) smaller change	1000	500	900	400	500	2.00	500	2.25
b) larger change	1000	500	600	100	500	2.00	500	6.00
Inconsistent: Decreasing absolute inequalities, constant relative inequalities								
a) smaller change	1000	500	800	400	500	2.00	400	2.00
b) larger change	1000	400	200	80	600	2.50	120	2.50
Consistent: Decreasing absolute inequalities, decreasing relative inequalities								
a) smaller change	1000	200	500	190	800	5.00	310	2.63
b) larger change	600	300	500	275	300	2.00	225	1.82
Consistent: Increasing absolute inequalities, increasing relative inequalities								
a) smaller change	1000	800	800	100	200	1.25	700	8.00
b) larger change	1000	500	900	300	500	2.00	600	3.00
