# Supplementary material for: Impact of Selective Evidence Presentation on Judgments of Health Inequality Trends: An Experimental Study
Source: PLoS One. 2013 May 16;8(5):e63362. doi: 10.1371/journal.pone.0063362 (PMC3656043; doi:10.1371/journal.pone.0063362)
Supplement: Table S2 — Logistic regression estimates for judgment of whether inequality increased or decreased. (PDF) [file pone.0063362.s005.pdf]

Table S2. Logistic regression estimates for judgment of whether inequality increased or decreased.

Variable	Estimate	Inconsistent Scenarios: Difference decreases, ratio increases	Inconsistent Scenarios: Difference constant, ratio increases	Inconsistent Scenarios: Difference decreases, ratio constant	Consistent Scenarios: Difference decreases, ratio decreases	Consistent Scenarios: Difference increases, ratio increases
Post vs. Pre	*β**	-0.373	0.000	-0.429	1.587	0.315
	95% CI	(-1.02,0.27)	(-0.70,0.70)	(-1.09,0.24)	(0.28,2.90)	(-1.34,1.97)
Ratio vs. Difference	*β*	0.114	-0.231	-0.000	0.830	-0.000
	95% CI	(-0.43,0.66)	(-1.52,1.05)	(-0.57,0.57)	(-0.42,2.08)	(-1.78,1.78)
Post X Ratio	*β*	1.865	1.484	2.281	-0.830	0.000
	95% CI	(0.96,2.77)	(0.32,2.65)	(1.21,3.35)	(-2.61,0.95)	(-2.14,2.14)
Large vs. small change†	*β*	0.538	0.451	-0.000	0.909	-0.000
	95% CI	(0.03,1.04)	(0.16,0.74)	(-0.47,0.47)	(-0.28,2.10)	(-0.88,0.88)
Constant	*β*	-1.012	-0.852	-0.302	0.993	2.197
	95% CI	(-1.72,-0.30)	(-1.73,0.02)	(-0.95,0.35)	(0.11,1.88)	(1.27,3.12)
Observations		160	160	160	160	160

*Log odds of agreement with ratio measure of inequality. CI, confidence interval (clustered by subject).
† p-values for treatment heterogeneity by Large vs. small change (i.e., Post X Ratio X Large) were 0.236, 0.152, 0.400, 0.573, and 0.471 across the 5 scenarios.
