# Supplementary material for: Impact of Selective Evidence Presentation on Judgments of Health Inequality Trends: An Experimental Study
Source: PLoS One. 2013 May 16;8(5):e63362. doi: 10.1371/journal.pone.0063362 (PMC3656043; doi:10.1371/journal.pone.0063362)
Supplement: Table S3 — Linear regression estimates for respondent’s judgment of program success. (PDF) [file pone.0063362.s006.pdf]

Table S3. Linear regression estimates for respondent's judgment of program success.

Variable	Estimate	Inconsistent Scenarios: Difference decreases, ratio increases	Inconsistent Scenarios: Difference constant, ratio increases	Inconsistent Scenarios: Difference decreases, ratio constant	Consistent Scenarios: Difference decreases, ratio decreases	Consistent Scenarios: Difference increases, ratio increases
Post vs. Pre	*β**	-0.125	-0.275	0.200	0.075	0.100
	95% CI	(-0.81,0.56)	(-0.63,0.08)	(-0.27,0.67)	(-0.30,0.45)	(-0.58,0.78)
Ratio vs. Difference	*β*	-0.650	-0.475	0.175	0.150	-0.225
	95% CI	(-1.07,-0.23)	(-1.13,0.18)	(-0.50,0.85)	(-0.36,0.66)	(-0.94,0.49)
Post X Ratio	*β*	-0.875	0.175	-1.525	0.275	-0.075
	95% CI	(-1.72,-0.03)	(-0.37,0.72)	(-2.39,-0.66)	(-0.27,0.82)	(-0.96,0.81)
Large vs. small change†	*β*	-1.512	0.087	0.763	1.512	-0.113
	95% CI	(-2.03,-1.00)	(-0.25,0.43)	(0.18,1.34)	(1.14,1.88)	(-0.56,0.34)
Constant	*β*	5.106	2.206	3.469	3.969	1.906
	95% CI	(4.35,5.86)	(1.66,2.75)	(2.93,4.01)	(3.47,4.47)	(1.41,2.40)
Observations		160	160	160	160	160

*Effect on subject's assessment of whether the hypothetical intervention was successful (1=not at all … 7=very successful). CI, confidence interval (clustered by subject). † p-values for treatment heterogeneity by Large vs. small change (i.e., Post X Ratio X Large) were 0.195, 0.768, 0.427, 0.373, and 0.379 across the 5 scenarios.
