# Supplementary material for: Impact of Selective Evidence Presentation on Judgments of Health Inequality Trends: An Experimental Study
Source: PLoS One. 2013 May 16;8(5):e63362. doi: 10.1371/journal.pone.0063362 (PMC3656043; doi:10.1371/journal.pone.0063362)
Supplement: Table S4 — Regression estimates for amount of money respondent was willing to give to support continuation of the hypothetical program. (PDF) [file pone.0063362.s007.pdf]

Table S4. Regression estimates for amount of money respondent was willing to give to support continuation of the hypothetical program.

		Inconsistent Scenarios			Consistent Scenarios	
		Difference decreases, ratio increases	Difference constant, ratio increases	Difference decreases, ratio constant	Difference decreases, ratio decreases	Difference increases, ratio increases
Post vs. Pre	*β**	3.45	-4.70	-4.65	-0.73	-9.05
	95% CI	(-10.21,17.11)	(-9.43,0.03)	(-11.73,2.43)	(-6.20,4.75)	(-14.95,-3.15)
Ratio vs. Difference	*β**	-12.60	2.60	1.92	7.92	-3.05
	95% CI	(-33.00,7.80)	(-8.71,13.91)	(-7.15,11.00)	(-1.22,17.07)	(-14.38,8.28)
Post X Ratio	*β**	-15.30	-5.67	-10.38	-0.63	3.63
	95% CI	(-32.48,1.88)	(-12.63,1.28)	(-22.05,1.30)	(-8.94,7.69)	(-4.12,11.37)
Large vs. small change	*β**	-28.00	16.59	26.16	26.49	12.14
	95% CI	(-47.56,-8.44)	(9.50,23.68)	(18.51,33.82)	(19.67,33.30)	(5.01,19.27)
Post X Large	*β**	-15.75				
	95% CI	(-34.40,2.90)				
Ratio X Large	*β**	16.55				
	95% CI	(-20.61,53.71)				
Post X Ratio X Large†	*β**	24.90				
	95% CI	(0.02,49.78)				
Constant	*β**	65.75	13.86	29.67	17.86	17.43
	95% CI	(51.97,79.53)	(6.29,21.42)	(21.50,37.84)	(9.35,26.36)	(9.05,25.81)
Observations		160	160	160	160	160

*Effect on amount of subject's money to be donated to support program continuation (Minimum=$0, Maximum=$100). CI, confidence interval (clustered by subject). † p-values for treatment heterogeneity by Large vs. small change (i.e., Post X Ratio X Large) were 0.050, 0.290, 0.052, 0.959, and 0.778 across the 5 scenarios.
