# Supplementary material for: Impact of Selective Evidence Presentation on Judgments of Health Inequality Trends: An Experimental Study
Source: PLoS One. 2013 May 16;8(5):e63362. doi: 10.1371/journal.pone.0063362 (PMC3656043; doi:10.1371/journal.pone.0063362)
Supplement: Table S5 — Regression estimates for respondent’s assessment of whether the hypothetical program should continue. (PDF) [file pone.0063362.s008.pdf]

Table S5. Regression estimates for respondent's assessment of whether the hypothetical program should continue.

		Inconsistent Scenarios			Consistent Scenarios	
		Difference decreases, ratio increases	Difference constant, ratio increases	Difference decreases, ratio constant	Difference decreases, ratio decreases	Difference increases, ratio increases
Post vs. Pre	*β**	-0.03	-0.20	-0.55	0.13	-0.43
	95% CI	(-0.66,0.61)	(-0.66,0.26)	(-1.30,0.20)	(-0.22,0.47)	(-0.87,0.02)
Ratio vs. Difference	*β**	0.07	-0.00	-1.05	0.15	0.37
	95% CI	(-0.34,0.49)	(-0.93,0.93)	(-2.02,-0.08)	(-0.36,0.66)	(-0.57,1.32)
Post X Ratio	*β**	-0.57	-0.83	0.65	-0.00	0.18
	95% CI	(-1.35,0.20)	(-1.48,-0.17)	(-0.44,1.74)	(-0.52,0.52)	(-0.42,0.77)
Large vs. small change	*β**	-1.31	1.29	0.95	1.58	0.84
	95% CI	(-1.72,-0.90)	(0.84,1.73)	(-0.00,1.90)	(1.18,1.97)	(0.32,1.36)
Post X Large	*β**			0.40		
	95% CI			(-0.58,1.38)		
Ratio X Large	*β**			1.00		
	95% CI			(-0.42,2.42)		
Post X Ratio X Large†	*β**			-1.80		
	95% CI			(-3.34,-0.26)		
Constant	*β**	6.01	3.21	5.00	3.99	2.88
	95% CI	(5.47,6.55)	(2.50,3.91)	(4.34,5.66)	(3.53,4.45)	(2.25,3.51)
Observations		160	160	160	160	160

*Effect on subject's assessment of whether the program should continue (1=definitely not … 7=very definitely should continue). CI, confidence interval (clustered by subject). † p-values for treatment heterogeneity by Large vs. small change (i.e., Post X Ratio X Large) were 0.369, 0.471, 0.024, 0.449, and 0.915 across the 5 scenarios.
